# Factors Related to the Implementation of Preconception Care Recommendations in Selected Districts of Limpopo Province: A Qualitative Study

**DOI:** 10.3390/healthcare11182586

**Published:** 2023-09-19

**Authors:** Ntombizodwa Paulinah Ndou, Thivhulawi Malwela, Maria Sonto Maputle, Ndidzulafhi Selina Raliphaswa, Lawrence Mabasa, Amidou Samie, Mutshinyalo Lizzy Netshikweta

**Affiliations:** 1 Faculty of Health Sciences, Department of Advanced Nursing Sciences, University of Venda, Private Bag X5050, Thohoyandou 0950, South Africa; zodwa.ndou@yahoo.com (N.P.N.); sonto.maputle@univen.ac.za (M.S.M.); ndidzulafhi.raliphaswa@univen.ac.za (N.S.R.); lizzy.netshikweta@univen.ac.za (M.L.N.); 2 Biomedical Research and Innovation Platform (BRIP), South Africa Medical Research Council, Tygerberg 7505, South Africa; lawrence.mabasa@samrc.ac.za; 3 Faculty of Sciences, Engineering and Agriculture, Department of Biochemistry and Microbiology, University of Venda, Private Bag X5050, Thohoyandou 0950, South Africa; samie.amidou@univen.ac.za

**Keywords:** recommendations, implementation, preconception care, perinatal outcomes

## Abstract

Preconception care (PCC) is the provision of biomedical, behavioural, and social health interventions to women and couples before they fall pregnant. The World Health Organization (WHO) developed PCC recommendations in 2013, which were included in the South African maternity care guidelines in 2016. The purpose of PCC is to lessen behaviours and environmental factors leading to maternal ill-health, thus reducing maternal and perinatal mortality rates. Objective: To determine the implementation of PCC recommendations at health facilities in the selected districts of Limpopo Province and the associated factors. Methods: A qualitative exploratory design was used. Nonprobability, purposive sampling was used to sample 29 professional nurses (PNs), and 51 women of childbearing age (WCBA) (19–35 years) from clinics and community health centres (CHCs). Data were collected through in-depth interviews with the professional nurses and focus group discussions with the WCBA. Data analysis was performed through open coding. Measures of trustworthiness were adhered to. Permission to conduct the study was obtained from relevant stakeholders, participation was voluntary and participants signed a consent form prior to data collection. Results: The findings of the study revealed that there was partial implementation of the PCC recommendations in the selected districts of Limpopo Province, PCC provision was dependent on clients’ initiation, a knowledge gap regarding PCC recommendations was identified from the professional nurses, and a lack of awareness regarding PCC from the WCBA. Conclusion: The preconception period is an important determinant of the pregnancy outcome; therefore, focus should be redirected to the pre-pregnancy period and not only to when the woman is already pregnant. However, to achieve this, professional nurses and WCBA should be empowered regarding PCC and its benefits.

## 1. Introduction and Background

Preconception care (PCC) is the provision of biomedical, behavioural, and social health interventions to women and couples before they fall pregnant [1]. The WHO developed PCC recommendations in 2013, which were adopted and included in the South African maternity care guidelines in 2016. The purpose of PCC is to lessen behaviours and individual environmental factors leading to maternal ill-health, thus improving perinatal outcomes. The components of PCC are risk assessment, health promotion, and medical and psychosocial interventions. PCC addresses a variety of areas—namely, nutritional status; genetic conditions, too early, unwanted and rapid successive pregnancies, environmental health, vaccines for preventable diseases, infertility/subinfertility, sexually transmitted infections, interpersonal violence, mental health, human immunodeficiency syndrome (HIV), female genital mutilation, psychoactive substance and tobacco use [1]. According to the recommendations, a woman who is intending to conceive within 12 months should be fully assessed and, if found to have any illness, treated and/or referred for further management or adjustment of existing chronic medications, genetic counselling performed if necessary and updating of vaccinations. The woman ought to be counselled about healthy living (diet, hygiene, exercise, rest, sleep and ensuring that any pre-existing conditions are controlled before she conceives) and the avoidance of risk factors (stress, alcohol, smoking, including secondary smoking, exposure to chemicals) and also offered folic acid supplements with the aim of preventing neural tube defects [1]. The aim of PCC is to ensure that a woman conceives while being healthy, thus leading to less complications during pregnancy. Several studies point out that the influencers of perinatal outcomes are multifactorial, e.g., a very young or advanced age, genetic factors, maternal malnutrition, lifestyle, infections and pre-pregnancy existing chronic conditions [2,3]. Many women tend to have poor pregnancy outcomes due to conditions that existed prior to pregnancy, such as hypertension, diabetes mellitus, and infections [4]. The above-mentioned conditions can lead to various poor pregnancy outcomes, for example, low birth weight, which is associated with poor nutrition, smoking, and hypertension, while miscarriages are associated with infections [5]. These are factors that should be dealt with or controlled during the preconception period because they can adversely affect either the mother or baby during pregnancy or post-delivery. It is important that a woman conceives when in a healthy state and not engaging in behaviours that may negatively affect pregnancy outcome [6,7]. Athrash and Jack [7] further pointed out that women on chronic medication who are planning pregnancy should be referred to a specialist for treatment adjustment or a change of treatment regimen before they fall pregnant. Ding et al. [6] asserted that people who receive PCC have higher chances of adopting a healthy lifestyle, having a planned pregnancy, receiving support from their partners and being likely to postpone pregnancy until they are older. Most high-income countries have started to implement the PCC recommendations, while middle- and low-income countries are lagging behind.

Nathan et al. [8] pointed out that non-implementation of PCC in Uganda was due to a lack of clear policy on PCC. In Malawi, implementation of PCC recommendations was low due to a lack of guidelines regarding PCC and a lack of knowledge by most health-care workers [9]. However, the authors further indicated there was an optimistic perception towards PCC by health-care workers and women of childbearing age [9]. In Masvingo, Zimbabwe, PCC was mostly provided in the private sector [10]. The same study further highlighted various barriers to PCC—namely, lack of knowledge amongst women, lack of funds for travelling to seek for PCC service, lack of support from spouses and peers, and the service not being prioritised by health-care workers [10]. A study conducted in KwaZulu-Natal in South Africa reported that although the health-care workers were well informed regarding PCC, they were struggling to implement the recommendations due to a lack of resources. In most cases, PCC was prioritised and offered only for clients with medical or surgical conditions and not to all WCBA due to a shortage of staff [11]. The same study also indicated that the health-care workers and women were not aware of the reproductive life plan.

The researchers observed that the PCC service in the selected districts of Limpopo Province was not offered. The emphasis in most health facilities was on the antenatal care service offered to pregnant women, with little or no effort being made to ensure that those who are not pregnant are in a healthy state before they conceive. There was information shared with clients, although it was not shared with the view of PCC. The researchers believe that the implementation of PCC can significantly reduce poor perinatal outcomes. This prompted the researchers to explore and describe the factors related to the implementation of PCC recommendations in selected districts of Limpopo Province.

## 2. Problem Statement

The perinatal outcomes in South Africa are still poor, with institutional neonatal data showing 12 deaths per 1000 live births, which has remained constant since 2018, while high-income countries are at 10/1000 live births [12]. Although there is increasing evidence to support the implementation of PCC recommendations, there is little evidence regarding the implementation of PCC recommendations in the selected districts of Limpopo Province, where the emphasis is still on antenatal care. This means that a number of women who might be suffering from various conditions may pass through the health facilities without being identified and treated until they fall pregnant. The other problem that was observed was that most pregnant women book late for antenatal care, which also affects the time for identification and prompt treatment of conditions they may be suffering from. The researchers were prompted to conduct a study on the factors associated with the implementation of PCC in the selected districts in Limpopo Province. The rationale for conducting the study was to determine what was happening in Limpopo in terms of the implementation of the WHO recommendations on preconception care, since Limpopo was among the top three out of nine provinces of South Africa in terms of perinatal mortality according to Statistics South Africa in 2018 [13].

## 3. Methodology

A qualitative approach employing an exploratory descriptive design was used from the 1st of April 2022 to 31st April 2023 to explore and describe the factors related to the implementation of PCC recommendations in selected districts of Limpopo Province. This method was chosen to obtain a richer understanding of the phenomenon under study.

The study setting was clinics and community health centres (CHCs) in the selected districts of Limpopo Province—namely, Capricorn, Mopani and Vhembe. The population comprised two categories of participants: 29 professional nurses who have been working for three or more years and 51 women of childbearing age (WCBA) (19–35 years) attending the clinic for maternal services or any other health-care service, whether having been pregnant before or not. Nonprobability, purposive sampling was used to sample the participants. Data were collected through unstructured in-depth interviews with 29 professional nurses and 8 focus group discussions (FGDs) with 6 to 8 members of the WCBA. Face-to-face interviews were conducted with the professional nurses in a quiet room in the various study settings as per arranged appointments. The question posed to the professional nurses was: “Could you explain your experience regarding the implementation of PCC recommendations in this health facility?” This question was followed up with probing questions (see Appendix A). The in-depth interview method was used with the professional nurses to allow for the collection of rich and extensive data from the nurses’ own speech and to observe language, which can provide insight into their intentions, feelings, and meaning. The researchers were nurses, making it possible for the researchers to build relationships with the participants and gain their trust, which can improve the data’s quality and richness. This method allows researchers the chance to follow up on and clarify participants’ responses, which can enhance the validity and dependability of the data. It enables researchers to modify and customize subjects and questions in accordance with participants’ requirements and interests, which can improve the data’s relevance and value. On the other hand, the focus group was conducted with the women as the researchers wanted to generate novel insights, observe the social interactions, and extract shared experiences, believing that synergistic influence can deepen the discussions and ensure the collection of rich data is achieved. The question for the FGDs was: “Can you explain what you understand by preconception care?” The questions were asked using the participant’s language to facilitate their understanding. This was also followed up by probing questions as guided by the responses from the participants (see Appendix B). The FGDs took place in a quiet room. The interviews and discussions were audio recorded, except when the participants were not comfortable to be audio recorded. The audio-recorded FGDs and in-depth interviews were transcribed verbatim. Field notes were also taken during the interviews. Data were initially collected from 23 professional nurses from 8 health facilities and 8 FGDs involving 33 WCBA from 6 health facilities, and when no new information was coming up, 6 more professional nurses and 2 more FGDs from another 2 health facilities were interviewed to ascertain if there will not be any other new information emerging. Thus, the sample size was estimated using the concept of data saturation. 

The interviewers were the researchers, who were not part of all the communities where the research was conducted even though they reside in one of the districts of Limpopo Province, because the study was carried in three districts of the province and in each district, there were number of clinics or community health centres included the study. The interviewers were the researchers themselves with the help of research assistants. All are conversant with conducting in-depth interviews as well as focus group discussions. All the interviewers possess the curiosity and open-mindedness to dig deep and pursue more information on the topic, are empathetic and connect with different personalities in the field of study, are conversant with multiple methods of data collection within qualitative studies and are ethical enough not to hurt or act unfairly or infringe the participants rights’ or to do harm rather than good. All were briefed prior to engaging in the activity of data collection. They all reside in Limpopo Province and know the languages spoken by the participants, thus they were able to interview the participants in their own language. Data were analysed and organized into themes and sub-themes using steps derived from Tesch’s descriptive data analysis method, whereby all the transcripts were carefully read to obtain a sense of the whole. One interesting interview transcript would be picked, read through and annotated on the margins as ideas surfaced in the mind. The researchers asked the following questions. What is it about? And what could be the underlying meaning? This task was completed for all the interviews. A list of all the topics was compiled, and similar topics clustered together. The columns for the major topics, unique topics, and leftovers were formed. The most descriptive words for the topics were found and turned into categories. Topics that related to each other were grouped by drawing a line to show interrelationships. A final category was made. Both the researchers and the independent coder analysed the transcripts independently. Themes and sub-themes regarding the implementation of PCC recommendations in the selected districts of Limpopo Province were developed. A literature control was performed to confirm the findings of the qualitative study [14]. 

## 4. Measures to Ensure Trustworthiness

The principle of credibility was ensured by prolonged engagement with the participants. The researchers met with the participants when going to the clinics or CHCs to make appointments for data collection. The researchers met with them on the set date for data collection and again maintained contact with them throughout while still analysing the data. The participants were again contacted to confirm whether what was captured was a reflection of what they said. Peer debriefing was performed to check other researchers’ views on the data collected. Persistent observation and member checks were used to enhance the credibility of the data [13]. To enhance transferability, the researchers selected information-rich participants through purposeful sampling. The researchers purposively used different locations—namely, clinics and CHCs in three districts—to enhance transferability. A thick description of the methods used in the study was produced to enable other researchers to decide whether the findings can be transferable to their own settings [15]. The dependability of the data was ensured through the use of a voice recorder. Cross-checking of the codes was performed by other research experts to see whether the experts would come up with codes such as those of the researchers [14]. To ensure confirmability, the researchers reflected on the data and bracketed their own views and beliefs about the phenomenon studied to avoid biases. The findings were sent to experts and promoters to confirm. The transcripts were independently coded by an independent coder to confirm whether the findings will be like those of the researchers [14,15,16].

## 5. Ethical Considerations

Permission to conduct the study was obtained from the University of Venda Research Ethics Committee (FHS/22/PDC/05/2504), the Limpopo Department of Health Ethics Committee, and district managers in the selected districts. The participation in the study was voluntary. Participants signed consent forms prior to data collection. The principles of autonomy, confidentiality and anonymity were adhered to throughout the study. The participants were informed of their right to participate without being coerced and their right to withdraw from the study whenever they feel like doing so without being penalized in whatever manner. Confidentiality was maintained by conducting the interviews in a private room. Anonymity was ensured by using codes instead of using the names of participants or the health facilities in which they were working [15].

## 6. Results

### 6.1. Demographic Characteristics of the Participants

Table 1, below depicts the number of Nurse’s interviews which was 29 participants. About 27 were females with ages ranging between 20–26 years. only 2 males with ages ranging from 40–50 years nursed formed part of the study.

Table 2, below presents the total number of participants who formed part of the 8 FGDs. The FGDs were homogeneous, meaning only women were recruited to form focus groups. The groups were representative of the three common languages in the province, Tshivenda, Sepedi, and Tsonga. Women between the ages of 19–25 were 17. 26–30 were 25 and 30–35 were 9 to make a total of 51 participants. The parity ranged between 0–2.

### 6.2. Findings from the Professional Nurses

This study found that there was partial and disorganized implementation of the PCC recommendations in the selected districts of Limpopo Province. The implementation depended on WCBA’s awareness of early initiation and preparation for conception or pregnancy. The associated factors were the knowledge gap regarding the PCC recommendations amongst professional nurses. Table 3 presents the results from the professional nurses and women of childbearing age.

#### 6.2.1. Theme 1: Implementation of PCC Recommendations

Two sub-themes emerged concerning the implementation of PCC recommendations—namely, partial implementation of PCC recommendations in most clinics, offered in an unstructured manner, and implementation of PCC dependent on the client’s initiation.

##### 6.2.1.1. Sub-Theme: Partial Implementation of PCC in Most Clinics, Offered in an Unstructured Manner

The findings of this study exposed that there were efforts made towards offering PCC in the selected districts of Limpopo Province. However, it was partial and not structured. The focus of PCC was more on women living with HIV (WLWHIV) instead of it being offered to all WCBA. This was evidenced by the following quotes: Professional nurse participant (PNP) no. 4 (PNP4), (PNP23), (PNP8): “…Mmm, we tell WLWHIV not to fall pregnant while the viral load is high.” (PNP17): “The HIV-positive clients are informed about the importance of treatment adherence, monitoring of viral load and postponement of pregnancy until the viral load is undetectable.” The study finding also revealed that although in some cases PCC was offered to women who were HIV-negative as well, it was not a full-service package, for example, no full assessment and/or supplements were given. Mostly, what was offered was health education to clients. This was demonstrated by the following verbal concessions from professional nurses from some clinics: (PNP23), (PNP1), and (PNP2): “…We teach clients about contraception, diet and healthy lifestyle.” The same sentiment was indicated by the clients, as evidenced by (WCBAP14): “Mmm… what usually happens eh… is that in the morning, while waiting to be attended to, we are taught about different stuff like what to eat to be healthy, and about contraceptives.” WCBA participant no. 40 (WCBAP40) had this to say: “Yeah, we are taught about the importance of taking our HIV treatment correctly and not to fall pregnant while the viral load is still high… but other than that is just monitoring of mmm… monitoring of the viral load and being taught about healthy taking diet and how to prevent reinfection.” Although the health education was provided, it was not focusing on WCBA per se and not viewed as part of PCC.

Even though the service was said to be offered, a full assessment was not performed and supplements were not given. This was evident from the reiterations by (PNP8, PNP7 and PNP23): “supplements, we do not give supplements to them, but when they report to be pregnant, we then give them supplements.” (PNP 27) had this to say: “… No, we usually do not give them any supplements unless the woman is anaemic, then we can give her ferrous sulphate.” (WCBAP 6) confirmed the above statements: “… I have never been given any supplements or any of those pills that we normally get when we are pregnant while not pregnant.” This is an indication that the PCC recommendations were partially implemented, mostly focusing on WLWHIV, who were also taught mainly with the aim of preventing mother to child transmission without any full assessment or supplements even when they indicated that they were planning to conceive. Therefore, many WCBA were not accessing any PCC service even though they regularly visited the clinics.

##### 6.2.1.2. Sub-Theme: Implementation of Preconception Care Dependent on Client’s Initiation

According to the guidelines, when consulting WCBA, the health-care worker should find out about their reproductive plan or intention to conceive in the near future. If the woman indicates that she is planning to conceive within the next 6 or 12 months or so, a full assessment is conducted, and if the woman is found to be having any condition, she is treated and given folic acid as a supplement. The woman would also be counselled about a healthy lifestyle and be informed about environmental risks she may be exposed to. Those with chronic conditions are supposed to be advised to postpone pregnancy until the condition is controlled, and some may need to be referred for adjustment of treatment or for genetic counselling. The findings of this study uncovered that the professional nurses expected their clients to volunteer information regarding their reproductive intentions in order to advise them on PCC. This was revealed by the following quotes: (PNP22): “… we do not get clients requesting for PCC service, actually we mostly see pregnant women. However, if they ask for it, we can offer them the service.” (PNP10): “…Clients only come when they are pregnant mmm…, they do not tell us they are planning to fall pregnant.” The above quotes suggest that professional nurses do not initiate a conversation with WCBA to find out about their reproductive intention and offer them PCC if they were planning to conceive. (WCBAP7) asserted: “I think the PCC you are talking about can be helpful because at times a person would like to be pregnant but does not know what to do so that the baby can be healthy, but I do not think women know that they can come to the clinic and get assistance on that regard.” The study discovered that in most of the clinics, WCBA were not aware of PCC, and the nurses who participated in the study indicated that they do not usually talk about PCC. This was affirmed by (PNP26): “… (sigh) hey, to be honest, this is an aspect that we do not cover when giving health education but from now onwards we will ensure that we teach our clients.”

#### 6.2.2. Theme 2. Knowledge Regarding PCC

The PCC recommendations have been included in the Guidelines for Maternity Care in South Africa since 2016. Thus, professional nurses should have been fully informed about the guidelines and also offer PCC services in the health facilities, and WCBA should have been aware of such a service and made use of it if they view it as important. The nurses need to inform women about the importance of being assessed before they conceive so that if there are any conditions or illnesses identified they can be treated or referred and also so that they can be given supplements such as folic acid. However, this was not the case. Therefore, knowledge regarding PCC was the second theme. One sub-theme was developed under this theme—namely, knowledge gap regarding PCC recommendations among amongst professional nurses.

##### 6.2.2.1. Sub-Theme: Knowledge Gap Regarding PCC Recommendations Identified from Professional Nurses

The findings of this study revealed that there was a knowledge gap on the part of the professional nurses regarding the PCC guidelines. In most instances, the participants just had an idea about what preconception care means prior to pregnancy, although the content of the PCC as a service was not known. While in other instances, the participants confessed that they knew nothing about PCC. This was evident by verbal concessions such as that from (PNP11) a primary health-care (PHC) trained professional nurse: “…what is it all about; I have never heard of that before, even during my PHC training I never heard of it [PCC].” (PNP3): “Mmh…, I have no experience, I know nothing about PCC.” These were confessions made by some professional nurse participants who indicated that they do not have any knowledge whatsoever regarding the PCC guidelines. Some of the professional nurses were of the idea that PCC was meant for people who were struggling to conceive, as evident by the following quote from (PNP8): “if a woman reports that she is struggling to conceive, we teach them about the menstrual cycle and indicate to them which days will they be more fertile mmh…, I mean when she is most likely to fall pregnant and if maybe she still fails to conceive, we refer her to the fertility clinic in hospital.” This was an indication that the participant lacked knowledge regarding PCC and thought it was indicated for people with subfertility.

### 6.3. Findings from the WCBA

#### 6.3.1. Theme 1: Awareness of PCC

Knowledge or awareness of a service enables a person to decide whether she/he has a need for it or not. Thus, it is important for a person to be aware of the availability of the service for her/him to use it. Lack of awareness may lead to the person’s failure to access the service at her/his disposal. Awareness of PCC was the only theme that emerged with one sub-theme—namely, the demonstration of a lack of awareness regarding PCC.

##### 6.3.1.1. Sub-Theme: Demonstration of a Lack of Awareness Regarding PCC

The findings of this study uncovered a lack of awareness about PCC as a concept and a service by most WCBA in all the health facilities in the selected districts of Limpopo Province. This was exposed by the following quote from (WCBAP5): “it is the first time I hear about it [PCC].”

After the researchers explained what PCC was all about, the participants still indicated that they had never heard about PCC and were not aware that they could, prior to being pregnant, go to the clinic and discuss with a health-care worker about their intention to fall pregnant, be assessed and be given supplement or be treated if found being ill or referred where required. (WCBAP12): ”I do not remember ever being taught or told about such a service or being offered such a service.” Some of the participants were indicating that they also believed that most women were not aware that they can go to the clinic before they become pregnant to receive any advice or be assessed, as expressed by (WCBAP13), who looked concerned: “… I don’t think most women know that they can come to the clinic to be taught about what they have to do or not to do while planning to be pregnant, or be checked if they were healthy, but I think if the service is well marketed people will use it.” This lack of awareness makes it difficult for clients to determine the importance of PCC or to utilize the service. Another participant (WCBAP4) had this to say: “…yeah I think if nurses can teach about this PCC like they do when teaching us on contraceptives or other things we can use it.” This demonstrates that although WCBA do not know about PCC, they view it as important and would consider using it if it is made available.

## 7. Discussion

This study found that there was partial implementation of the PCC recommendations in most clinics. PCC was mostly offered to WLWHIV to prevent mother to child transmission, with many other services of PCC being not offered. WLWHIV were educated about the importance of postponing pregnancy until their viral load was controlled or not detectable. The women’s viral load would be closely monitored, and they were also told about the importance of adherence to treatment and use of condoms to prevent reinfection and disclosure to their partners. Guttin et al. and Khonje [17,18] obtained similar findings in Botswana and Malawi, respectively, where the focus was only towards WLWHIV. This could be probably due to the fact that there are protocols regarding the management of fertile seropositive clients while there are no clear guidelines regarding PCC. The study also found that the PCC services offered were mainly health education on diet, contraceptives and hygiene, which was given generally to all patients and not specifically to clients requesting PCC or to WCBA. The results also revealed that general assessment, genetic counselling or referral for genetic service, risk assessment, referral to the doctor for adjustment of treatment and giving of supplements was not performed. These findings are similar to the findings of Ojifinni and Ibisomi [19], where it was found that most clients, even those with chronic conditions such as hypertension, were not offered PCC even though it was indicated. Women who were advised about the importance of postponing pregnancy until their viral load was low or undetectable were not given any supplements. Most of the professional nurses who participated in the study pointed out that supplements were indicated when women were pregnant. Folic acid helps to prevent neural tube defects during the foetal developmental stage [20]. The importance of folic acid during the preconception period was overlooked or not known by the professional participants in this study. Stephenson et al. [5] argued that folic acid given to women during pregnancy helps to improve any micronutrient deficiency the mother might be suffering from but does not have any effect to the foetus, who would have benefited if the folic acid was given to the woman before conception. Inconsistent assessment of clients during the preconception period means that many problems are left unidentified and unattended to. Some of the problems may lead to complications and result in poor perinatal outcomes [20]. According to Jamee, Sen and Bari [21], perinatal outcomes are determined before the woman falls pregnant. The findings of the study exposed gaps in the care offered to clients with other chronic conditions besides HIV. In most of the clinics, there was very little done for clients suffering from other chronic conditions and no adjustment of treatment during the preconception period. The participants in this study indicated that women with chronic conditions were referred to high-risk clinics when they are pregnant. Ojifinni and Ibisomi [19] supported the above findings.

The findings of this study revealed that the implementation of PCC recommendations was dependent on WCBA’s initiation. The professional nurses were not initiating the PCC conversation, so in most cases, if the WCBA did not request it, they were not offered PCC. The one key question: “Do you have intentions to conceive in the next year?” was not used to find out about clients’ reproductive intentions even though they were of a childbearing age. The professional nurses expected their clients to volunteer information regarding their intention to conceive for them to give advice to the patient. Ukoha and Mtshali [11] confirmed these findings. The aforementioned key question used to find out about the WCBA’s reproduction intention in the following year assists in opening up a PCC discussion. Should the woman say she is not planning any pregnancy soon, she is encouraged to use contraceptives to prevent unplanned pregnancy. If the woman’s response is that she is planning to conceive in the near future, a full assessment is performed and, if found to be having any illness, she ought to be treated and/or referred for further management and given health advice about healthy living and risk factors to avoid [1]. The professional nurses who participated in this study were of the idea that women should be the ones who request the service or indicate that they are intending to conceive, and since women were not indicating their reproductive intentions, the service was not offered. However, PCC should be offered to all WCBA in order to enhance maternal health, thus reducing complications that can lead to poor perinatal outcomes [1]. This means that most WCBA do not receive PCC service, especially since they were not aware that there is such a service and consequently were not requesting it, as this study discovered [17,22]. A knowledge gap regarding PCC among the professional nurses seemed to be the problem leading to the poor implementation of the PCC recommendations. The professional nurses who participated in this study had scant knowledge about the full-service package of PCC, as some were not sure when to offer such a service or to whom it was supposed to be offered. Some viewed it as a service that was supposed to be exclusively offered to women who could not fall pregnant and WLWHIV. These women would only be referred to infertility clinics. Most WCBA were not offered the service because the professional nurses were not aware that all WCBA were supposed to receive the service. In fact, most of the participants were not very clear of the PCC recommendations. These findings are similar to those of Abayneh et al. [23], who found that health-care workers were not knowledgeable regarding PCC. Kasa et al. [24] asserted that a lack of the required knowledge makes it difficult for a person to do what may be expected of him/her. The PCC recommendations were developed in 2013 by the WHO and incorporated into the maternity guidelines in South Africa in 2016, although the professional nurses were not well informed about them. This knowledge gap influenced their ability to offer PCC to clients. A study by Ololande et al. [25] demonstrated correlation between the level of knowledge and the practice of PCC. Professional nurses in CHCs and clinics are well positioned to provide the PCC service to WCBA to improve the perinatal outcomes for the mother and the neonate. Their role in this service is to communicate unambiguous, correct, and appropriate information, screen for and act on potential barriers to a successful perinatal outcome as well as refer clients appropriately where necessary. However, this was not possible due to the knowledge gap identified in this study.

This study exposed that the WCBA lacked knowledge regarding PCC and were not aware of the PCC services that can be offered at the clinics and/or CHCs. Awareness of something generates an interest to access it if one perceives it as important. WCBA could only be aware of PCC if they were informed about it and the benefits thereof at the clinics or CHCs that they usually visit for various reasons. Since most WCBA were not aware of PCC, they could not make an informed decision or determine its importance. The lack of awareness by WCBA about PCC also affects their utilization of the service. On the other hand, the professional nurses expected WCBA who were not aware of PCC to request it. Lack of awareness has a negative influence on the aim to improve perinatal outcomes. The researchers believe that if WCBA are made aware of PCC, most are likely to utilize it and thus improve their perinatal outcomes. The findings of this study echo those of Woldeyohannes [26] and Okemo et al. [27], who also found that most WCBA were not aware of PCC and thus likely not to use the service. A study by M’hamdi et al. [28] also concluded that a lack of awareness of PCC is a barrier to its utilization. There is therefore a need to improve the awareness of community members, especially WCBA, regarding PCC to increase the uptake of PCC.

## 8. Strengths and Limitations of the Study

The strength of this study was that data were collected from professional nurses and WCBA. Data were collected from three districts in the province. This enabled the researchers to obtain a reflection of what was happening about the implementation of the PCC recommendations in the selected districts of Limpopo Province. Although the findings contribute to the field of study, they are limited to the districts where the study was conducted. 

## 9. Recommendations

In-service training and workshops for professional nurses should be held concerning the PCC guidelines so that they can be empowered with knowledge and skills and be able to implement the guidelines. The PCC should be integrated into the services that are in place and be viewed as a continuum in maternal care. Furthermore, health education and awareness campaigns should to be conducted to raise awareness of PCC in the community and to make it accessible to WCBA. The researchers also recommend that strategies for the implementation of the PCC recommendations in the selected districts be formulated so that WCBA can have access to this important service.

## 10. Conclusions

The results of this study demonstrate the partial implementation of the PCC recommendations, which can be related to the knowledge gap among professional nurses and the lack of awareness among WCBA. The preconception period is an important determinant of the pregnancy outcome; therefore, a mindset shift from the traditional norm that defines the onset of pregnancy as the starting point for attention to neonatal health is needed.

## Figures and Tables

**Table 1:** Demographic characteristics of the professional nurses.

No. of Professional Nurses Interviewed	Gender	Age
	Females = 27	20–30 = 6
	Males = 2	40–50 = 14
		>50 = 9
Total	29	29

**Table 2:** Demographic characteristics of the WCBA.

Number of FGDs	Age	Parity	Language
**8**	19–25 = 17	0 = 3	Sepedi = 19
	26–30 = 25	1 = 8	Tshivenda = 15
	30–35 = 9	2 = 2	Tsonga = 17
Total	51	51	51

**Table 3:** Results categorized as themes and subthemes from professional nurse participants (PNPs) and women of childbearing age (WCBA).

No.	Themes	Sub-Themes
6.2.1.	Implementation of PCC guidelines	6.2.1.1. Partial implementation of PCC recommendations in most clinics, offered in an unstructured manner
		6.2.1.2. Implementation of PCC dependent on client’s initiation
6.2.2.	Knowledge regarding PCC	6.2.2.1. Knowledge gap regarding PCC recommendations identified from professional nurses
**Themes and sub-themes from women of childbearing age (WCBA)**		
6.3.	Awareness of PCC	6.3.1.1. Demonstration of a lack of awareness regarding PCC.

## Data Availability

Data are available on request.

## Appendix A. In-Depth Interview Guide

The following questions will lead the discussion/interview:

- Can you share your experience with the implementation of preconception care in this health facility?
- In your view, what could be obstacles to the implementation of preconception care in this health facility (if any)?
- What do you think can be done to strengthen the implementation of preconception care in this health facility?

These were the main questions that were followed-up by probing questions that emanated from the responses of the participants.

## Appendix B. Interview Guide for a Focus Group Discussion with Women of Child Bearing Age

- Can you explain what you understand by preconception care?
- Which factors will encourage women to utilize preconception care services?
- What challenges/factors will limit women aged 19–35 years using these services?
- What suggestions/recommendations would you give concerning preconception care services in this facility?

These were the main questions that guided the focus group discussions. Follow-up probing questions were asked, emanating from the responses from the participants.
